# Lightweight CoAP-Based Bootstrapping Service for the Internet of Things

**DOI:** 10.3390/s16030358

**Published:** 2016-03-11

**Authors:** Dan Garcia-Carrillo, Rafael Marin-Lopez

**Affiliations:** Department Information and Communication Engineering (DIIC), Faculty of Computer Science, University of Murcia, Murcia 30100, Spain; rafa@um.es

**Keywords:** IoT, bootstrapping, CoAP, EAP, AAA, lightweight

## Abstract

The Internet of Things (IoT) is becoming increasingly important in several fields of industrial applications and personal applications, such as medical e-health, smart cities, *etc*. The research into protocols and security aspects related to this area is continuously advancing in making these networks more reliable and secure, taking into account these aspects by design. *Bootstrapping* is a procedure by which a user obtains key material and configuration information, among other parameters, to operate as an authenticated party in a security domain. Until now solutions have focused on re-using security protocols that were not developed for IoT constraints. For this reason, in this work we propose a design and implementation of a lightweight bootstrapping service for IoT networks that leverages one of the application protocols used in IoT : *Constrained Application Protocol* (CoAP). Additionally, in order to provide flexibility, scalability, support for large scale deployment, accountability and identity federation, our design uses technologies such as the *Extensible Authentication Protocol* (EAP) and *Authentication Authorization and Accounting* (AAA). We have named this service CoAP-EAP. First, we review the state of the art in the field of bootstrapping and specifically for IoT. Second, we detail the bootstrapping service: the architecture with entities and interfaces and the flow operation. Third, we obtain performance measurements of CoAP-EAP (bootstrapping time, memory footprint, message processing time, message length and energy consumption) and compare them with PANATIKI. The most significant and constrained representative of the bootstrapping solutions related with CoAP-EAP. As we will show, our solution provides significant improvements, mainly due to an important reduction of the message length.

## 1. Introduction

Over the last few years, the global information network formed by Internet-connected objects known, as the Internet of Things (IoT) [1] has experienced an impressive growth. To accomplish the vision of the Internet of Things, standardization organizations and the research community have been working on the definition of several architectures and protocols [2,3].

These networks lead the trend that every device is connected to the Internet and can exchange information. Indeed, an important part of the IoT networks is foreseen to be formed by a vast amount of devices with constrained capabilities (*smart objects*) and IP-based networking connectivity [4]. These are typically based on low power radio technologies [4] such as IEEE 802.15.4 [5] or Bluetooth Smart [6]. For IP-based communications, the IP version *6 over Low power Wireless Personal Area Networks* (6LowPAN) standard [7] enables IPv6 connectivity for smart objects. This brings in new and promising areas of application such as smart cities, smart grids, home automation, e-healthcare, among others. While it allows for opportunities and improvements in our daily life, it also raises multiple security risks, which need to be tackled.

In particular, one of the basic aspects of security is the *bootstrapping* of the smart object [8]. As described by Garcia-Morchon *et al.* [9] bootstrapping refers to the process by which a smart object securely joins the IoT network at a given time and location. It includes the *authentication* and *authorization* of a device as well as the transfer of security parameters (e.g., keying material) to the device allowing a trustworthy operation in a particular network. As a consequence of the process, the node joins a security domain and *accounting* of the usage of the bootstrapping service may be carried out. In very demanding IoT scenarios, such as smart cities, a high number of smart objects will need to be authenticated and authorized before joining a security domain. The bootstrapping service is in charge of these operations. Additionally, these smart objects may belong to different organizations but may be deployed and bootstrapped in the same security domain. Thus, the concept of identity federation becomes relevant, though it has not yet been clarified how it will be managed [10].

In this sense, we foresee the key importance of *Authentication, Authorization and Accounting* (AAA)-based infrastructures [11] to provide a flexible, scalable and federation-aware bootstrapping service in IoT. The reason is two-fold: first, they are robust infrastructures for managing the authentication, authorization and accounting of the activity the smart objects and, in conjunction with the *Extensible Authentication Protocol* (EAP) [12], provide a secure framework for flexible authentication, authorization and key distribution [13,14]. Some evidences of the use of AAA framework in the context of IoT can be found in [15,16,17]. Second, it is widely used to manage a great number of device connections and, therefore, AAA support large scale deployments. In fact, AAA infrastructures based on the protocol Diameter [18] are commonly used in 3G networks to control the access of millions of users [19]. Another example is *eduroam* (educational roaming network) [20], which is a world-wide federation for WiFi connectivity across campuses and research and educational organizations around the world, that support thousands of users. Eduroam deploys EAP and an AAA infrastructure based on the *Remote Authentication Dial In User Service* (RADIUS) [21], which provides the identity federation substrate.

In this context, we propose a novel bootstrapping service that is built on top of the *Constrained Application Protocol* (CoAP) [22] with the assistance of EAP and AAA infrastructures. The main reason for using CoAP is that it has recently been standardized as the application protocol for exchanging information between smart objects and, therefore, is specifically designed for devices with small memory and computational resources, such as those expected in IoT networks. In fact, our proposal stems from the (realistic) assumption that the smart objects will generally ship a CoAP implementation. Our service also assumes the presence of a centralized entity, the *controller* (such as, e.g., the coordinator in ZigBee IP [23]) which manages the access to a particular security domain and interacts with a smart object to perform the bootstrapping. That is, the controller authenticates and authorizes the smart object to become a member of the security domain.

To achieve this, both entities use CoAP to transport EAP packets for the authentication and to carry authorization. The controller can interface with a backend AAA infrastructure to complete the EAP authentication, perform the authorization steps related with the bootstrapping service and, optionally, account for the activity of the smart object in the security domain.

In this paper, we present our bootstrapping service, named *CoAP-EAP*, its architecture, the design and the performance evaluation with implementation in the Contiki OS Cooja simulator [24], as well as comparison with PANATIKI [25], which represents the best case of bootstrapping solutions also using EAP and AAA. The rest of this paper is divided as follows. Section 2 gives the background and state of the art, in order to understand our solution. Section 3 details the proposed bootstrapping service architecture and operation. In Section 4, we show performance evaluation including message size, bootstrapping time, memory footprint, the probability of finishing a bootstrapping (success percentage) and energy consumption. Finally, we provide some conclusions and future work lines in Section 5.

## 2. State of the Art

### 2.1. Authentication, Authorization and Accounting (AAA) Framework

The *Authentication, Authorization and Accounting* (AAA) Framework [11] provides support for the three basic security services in network deployments: authentication (to determine who the end user is), authorization (to determine under what conditions an end user is granted access to the network resource), and accounting (to register the resource consumed by the end user). Thus, it is consistent with at least two of the required processes, authentication and authorization, involved during the bootstrapping.

The AAA framework defines a model consisting of: an *End User* (EU) desiring access to a specific network service; an *Identity Provider* (IdP) which has registered the end user’s identity and long-term credentials (e.g., a certificate or pre-shared key); and a *Service Provider* (SP) operating and controlling the access to the network service. In a *non-federated case*, the IdP and SP belong to the same organization (*IdP’s organization*). In *federated cases*, where some bilateral agreements are assumed among different domains joining the federation, the IdP and SP belong to different organizations that operate independent AAA servers: *the IdP’s AAA server* and *the SP’s AAA server*. Both AAA servers are capable of exchanging authentication, authorization information and accounting data.

Additionally, the SP also operates the entity that intermediates between the smart object and the AAA infrastructure to carry out the authentication and authorization processes. This entity is generally called *Network Access Server (NAS)*. Thus, the simplest AAA infrastructure consists of a NAS directly connected to a AAA server by a AAA protocol. Nevertheless, several AAA servers (usually called *AAA proxies*) can be deployed between the NAS and the AAA server for scalability reasons or federated access support.

Either way, the NAS, AAA proxies and AAA servers that constitute the AAA infrastructure exchange information by using AAA protocols. Today, the most commonly deployed AAA protocols are RADIUS [21] and Diameter [18]. Although the latter is the most complete, in terms of the functionality provided by its design, RADIUS is still one of the most widely deployed protocols within existing AAA infrastructures [26].

The *Remote Authentication Dial In User Service* (RADIUS) [21,27] is a client-server protocol where a NAS acts as RADIUS client transporting messages on top of UDP. The RADIUS client sends AAA information in a RADIUS message request to the RADIUS server, which answers with a RADIUS response. There can be two RADIUS servers for different purposes: the authentication and authorization server (RFC 2865) that keeps a database with registered users and accounting server (RFC 2866) that collects data from the service usage (data sent and received, connection time, *etc.*).

In contrast, Diameter [18] is an evolution to solve some of the RADIUS limitations in terms of scalability and security. It uses a reliable transport like TCP or SCTP [28]. For the security part, it uses either IPsec [29] or TLS [30] and provides a message format allowing a longer message size and a higher number of concurrent connections. Thus, it is better adapted for multiple numbers of end users.

### 2.2. The Extensible Authentication Protocol (EAP)

The *Extensible Authentication Protocol* (EAP) [12] allows different types of authentication mechanisms (e.g., based on symmetric keys, digital certificates, *etc.*) named *EAP methods*. For example, EAP-PSK [31] is an EAP method based on the use of a pre-shared key (PSK) to provide a lightweight authentication mechanism. Another examples of EAP methods can be found in [32].

EAP is a lock-step protocol, which supports only a single packet, request or response, in flight. Each request message (*EAP Request*) is answered with a response (*EAP Response*). The number of exchanges will depend on the EAP method selected. Every EAP method runs between the *EAP peer*, and the *EAP server* through an *EAP authenticator*. From a security standpoint, the EAP authenticator acts as a mere EAP packet forwarder.

To perform an EAP authentication, the EAP authenticator usually starts the process by requesting the EAP peer’s identity through an EAP Request/Identity message. The EAP peer answers with an EAP Response/Identity with its identity. The identity follows the *Network Access Identifier* (NAI) format [33] (e.g., *smartobject@domain*). It contains a smart object’s identity information (*i.e.*, *smartobject*) separated with an @ and the domain (*i.e.*, *domain*) it belongs to. With this information, the EAP server will select the EAP method to be performed. The EAP method execution involves several EAP Request/Response exchanges between the EAP server and the EAP peer.

**Figure 1:**
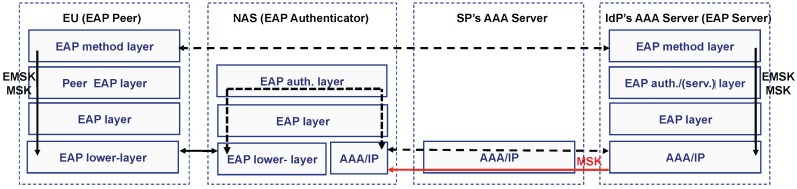
Extensible Authentication Protocol (EAP) pass-through model.

There are two deployment models of deployment for EAP. On the one hand, the *standalone EAP authenticator model*, where the EAP server is co-located in the same device as the EAP authenticator. This may be appropriate in deployments with a small number of objects. In this case, there is no AAA infrastructure in the backend. On the other hand, there is the *pass-through EAP authenticator* model, which is the most scalable configuration. In this model, the EAP server and the EAP authenticator are implemented in separate nodes. Specifically, the EAP server is centralized on an AAA server deployed somewhere on the Internet and gives service to several EAP authenticators. Here, the communication between the EAP server and the pass-through EAP authenticator is performed using an AAA protocol. In both cases, a protocol referred to as the *EAP lower-layer* is used to transport the EAP packets between the EAP peer and the EAP authenticator. As we will see in Section 3, CoAP-EAP defines a new EAP lower-layer by using CoAP messages. Figure 1 shows a mapping between the entities defined in the AAA model and EAP pass-through model. Each layer in the EAP processes a part of the EAP message in each entity, as described in [12]. As observed, the EAP method is processed in the EAP peer and the EAP server in the IdP’s AAA server.

Certain EAP methods [32] are able to generate keying material. Specifically, according to the *EAP Key Management Framework* (EAP KMF) [14], two keys are exported after a successful EAP authentication: the *Master Session Key* (MSK) and the *Extended Master Session Key* (EMSK). The first is traditionally sent to the authenticator to establish a security association with the EAP peer, while the second must not be provided to any other entity outside the EAP server and peer. Thus, both entities may use the key material for further purposes.

Recently, the applicability statement defined in [12], which limited EAP for network access authentication, has been enriched to allow the use of EAP in application access authentication [34].

### 2.3. The Constrained Application Protocol: CoAP

Bormann *et al.* [35] discuss the possibility of smart objects being susceptible to offering some resources or services represented by an *Uniform Resource Identifier* (URI). While this is solved in non-constrained deployments with HTTP. In IoT, instead of HTTP, the *Constrained Application Protocol* (CoAP) [22] has been proposed as a web transfer protocol based on the REST [36] model. The main reason is that CoAP has been specially designed for constrained devices with shorter message length and less demand on resources. In fact, HTTP might be a too weighty protocol for some types of smart objects. As analyzed in [35], HTTP poses a considerable implementation burden that exceeds the capabilities of small devices. Indeed, IoT devices [37] are constrained in memory, energy and computational resources and are expected to be deployed in constrained networks, where increasing the message length by a few bytes of information might result in fragmentation in the links. A constrained application protocol as CoAP, with short message length and low computational requirements, therefore alleviates these problems. For all these reasons, although CoAP has certain similarities with HTTP, they are not compatible.

It is worth noting that the transport of CoAP messages in IEEE 802.15.4e frames is currently being considered [38]. CoAP has been designed with several features, of which we highlight: (1) low overhead and low parsing complexity; and (2) support for the discovery of CoAP resources and services. On the one hand, low overhead mainly refers to the simple message format and protocol exchange that leads to a reduced parsing and processing complexity, saving system resources as a consequence (CPU, memory, battery, *etc*.). On the other hand, the discovery of CoAP services and resources is also important since it is expected that the number of devices and services offered by these devices will grow rapidly [1]. Additionally, UDP binding in CoAP provides optional reliability when supporting unicast and multicast requests.

CoAP defines an *endpoint* as an entity that participates in a CoAP exchange. A CoAP message has a 4-byte header and optionally a set of options and a payload. Each message contains a *Message ID* used to detect duplicates and for optional reliability. A message can be of a type (Type field) *Confirmable* (CON), *Non-confirmable* (NON), an *Acknowledgement* (ACK) or a *Reset* (RST). CON or NON messages can be *requests* or *responses* depending on the *Code field* value in the header. An endpoint sending a Confirmable message will first wait for an *Acknowledgement* (ACK) message. To improve the efficiency (by sending more information with fewer messages), a *piggybacked response* can be included in the payload of an ACK to answer a Confirmable request. On the contrary, if a Non-confirmable message is sent, an ACK message is not expected.

A *CoAP client* is an endpoint that sends *requests* to a *CoAP server* for a service. When the CoAP server receives a request, it may send a response. A token value (*Token*), which is chosen randomly (it could have zero length) is used to relate a request with the corresponding response.

If the CoAP server cannot process a message, it will send a RST message to indicate the inability to process it. This might happen, for example, when the smart object reboots and has forgotten some state to process the message. The RST can also be used as for the aliveness test of an endpoint, also called *CoAP Ping*. If the server is going to answer a Confirmable message with a CoAP response, but the information is not available yet, it can send an ACK message with empty payload (a CoAP message containing only the header) to indicate that the response will arrive later.

CoAP defines four basic request methods *GET*, *POST*, *PUT* and *DELETE*. The client can use the GET method to retrieve information from the server. It can also use the POST method to create a new resource in the server. The server will then assign an identifier to the created resource. From that point, the client can use the POST or PUT method to update it. Finally, DELETE is used to erase a resource in the server.

### 2.4. General Concept of Bootstrapping

The term bootstrapping is not new. It was coined in the early 1950s to refer to a load button that was used to initiate a bootstrap program or small program to start the operating system. In computer communications, one of the first protocol to use the term bootstrap was the *Bootstrap Protocol* (BOOTP) [39] to provide an IP address to a communicating host. This term has evolved, incorporating security aspects and more recently it is referred to in the literature as the process by which a node gathers any necessary information (not only an IP address but also security parameters) to join a network or a service after an authentication and authorization processes [40,41]. That information can be, for example, available cipher-suites, shared keys, certificates, service parameters, *etc*. Different services can instantiate a bootstrapping solution tailored to their specific needs.

For example, Mobile IPv6 (MIP6) considered as a service, requires dynamic parameters (cryptographic material, home address IPv6 configuration, *etc.*) to be configured for its correct operation [42]. This can be done dynamically after a bootstrapping for the MIP6 service such as that defined in [43], where the *Extensible Authentication Protocol* (EAP) [12] and *Internet Key Exchange v2* (IKEv2) [44] have been selected to carry out this process. Another example is related to *Dynamic Host Configuration Protocol* (DHCP) authentication extension [45] where some cryptographic material is required to protect DHCP. A bootstrapping solution proposed in [46] provides the required material by using EAP transported over an extension of DHCP. As a final example, 3GPP also defines a *Generic Bootstrapping Architecture* (GBA) [47] to tackle the bootstrapping problem in cellular networks.

**Figure 2:**
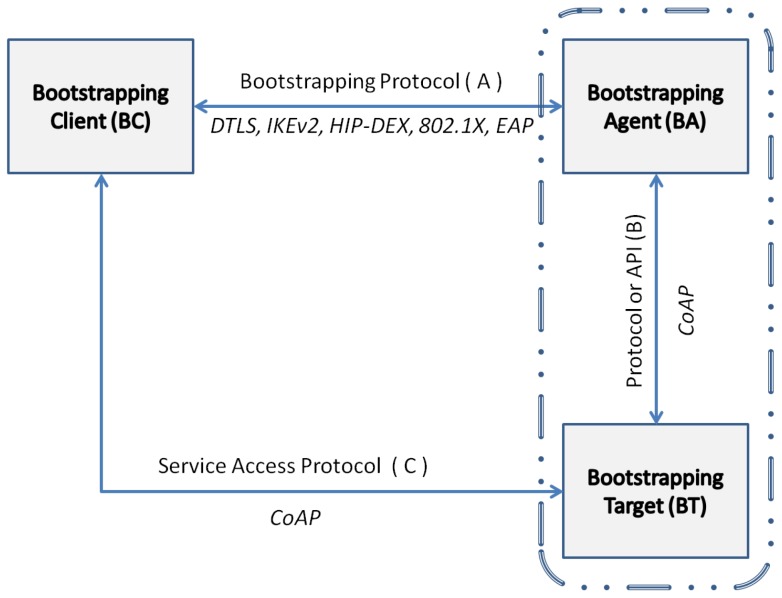
Bootstrapping framework for internet of things (IoT).

In general, the concept of bootstrapping can be seen as a framework to be instantiated in different scenarios. As explained in [48] there are three entities involved. A *Bootstrapping Client* (BC), *Bootstrapping Agent* (BA) and *Bootstrapping Target* (BT). Figure 2 illustrates the framework. The BC is the entity asking for access to a service. The BA manages the resources and services, authorizing the access to those services. The BA can rely on an external entity called Authentication Server (AS) to complete the authentication and authorization of the BC. This is what we will do in the design of our architecture (see Section 3.2). The BT is the entity in charge of providing the service under the direction of the BA. The BA and BT can be co-located in the same entity or different entities depending on the deployment.

There is a protocol or interface, *A*, used between BA and BC, so they can exchange information about the access to a specific service. Another interface called *B* serves to communicate both the BA and the BT and to configure under what conditions BC will access the services offered by BT. The interface *C* is used between the BC and BT, for BC to request access to the service.

As we will detail in Section 2.5, in the particular case of IoT, the interface *A* can be instantiated by using *Datagram Transport Layer Security* (DTLS) [49], *HIP Diet EXchange* (HIP-DEX) [50], IKEv2 [44], *IEEE 802.1X* [51], or EAP [12]. *B* might not be necessary if BA and BT are co-located, but if they are separated entities, the protocol to communicate that BC has permissions to access the service may be CoAP. *C* can be instantiated with CoAP as application protocol to request access.

### 2.5. Bootstrapping in IoT

T. Heer *et al.* [15] and Garcia-Morchon *et al.* [9] describe the concept of bootstrapping in IoT as the process of a smart object securely joining an IoT network at a specific place and time. It includes the *authentication* and *authorization* of the smart object as well as the transfer of some security parameters (e.g., keying material), so allowing a trustworthy operation in a particular domain. As a consequence of the process, the smart object joins a security domain and *accounting* of the usage of the bootstrapping service may be carried out. The bootstrapping process is specially important in the case of IoT where the configuration of the smart objects is expected to be as much automated as possible, making the scalability of the deployments easier.

In general, the importance of bootstrapping in IoT has been highlighted in several works [52,53]. On the one hand, the problem of bootstrapping has been discussed in several IETF Working Groups (WG) such as *IPv6 over the TSCH mode of IEEE 802.15.4e* (6tisch), *Authentication and Authorization for Constrained Environments* (ACE) WG, *Constrained RESTful Environments* (CoRE) WG and *IPv6 over Networks of Resource-constrained Nodes* (6lo) WG. Additionally, an IETF mailing list has been created specifically to discuss the bootstrapping [54]. Other standardization organisms and alliances as IEEE, W3C, OMA, ETSI and IPSO among others, are working in IoT and some of them provide insights into the bootstrapping process in IoT.

On the other hand, there are several proposals discussing the general problem of bootstrapping for constrained devices while others also propose solutions that consider the use of EAP and, in some cases, the interaction with AAA infrastructures.

Garcia-Morchon *et al.* [9] analyze of the IP-based security protocols for bootstrapping in IoT networks. In the case of a centralized architecture, as our bootstrapping service, they highlight the potential use of EAP as a protocol to perform authentication and generation of fresh keying material. *The Protocol for carrying Authentication for Network Access* (PANA) [55] is proposed as a candidate to transport EAP between the smart object, acting as the *PANA client* (PaC), and the controller, which is the *PANA agent* (PAA) in PANA terminology. Nevertheless, these authors also recognize that the transfer of configuration parameters in a centralized scenario can be made by other protocols. In our solution, CoAP is used instead of PANA as EAP lower-layer.

O’Flynn *et al.* [56] discuss the general problem of bootstrapping for low-power wireless networks. They also consider a centralized architecture with a root trusted entity. They consider the option of EAP as authentication protocol and analyze PANA and IEEE 802.1X [51] as possible EAP lower-layers. However, as analyzed in [57], link-layer solutions, such as IEEE 802.1X, are unsuitable for multi-hop wireless networks. Additionally, He *et al.* [53] includes the possibility of using HIP-DEX [50] as a bootstrapping protocol, although this option does not have any interaction with EAP or AAA’s, limiting the case to small or medium scenarios. Nevertheless, no concrete alternative is chosen in these works.

Sarikaya [58] and Sarikaya *et al.* [59] propose the use of EAP-TLS, based on certificates, as a specific method for authentication during the bootstrapping. The authors also consider PANA or IEEE 802.1X as EAP lower-layers.

S. Das *et al.* [16] propose a centralized alternative using PANA, EAP and AAA to bootstrap a pre-shared key (PSK) to establish a DTLS [49] or IKEv2 [44] unicast security association between the smart object and the PAA (the controller). However, CoAP is used afterwards for the post-bootstrapping phase. In particular, the CoAP client (the smart object) requests the CoAP server to bring a key (*pull model*) from an *Authentication Server* (AS) which acts as EAP server (*i.e.*, AAA server) in the EAP authentication involved in the bootstrapping. Our solution does not require a specific protocol just for bootstrapping (PANA) but reuses the deployment of CoAP to build the bootstrapping service.

Moreno *et al.* [25] designed and implemented a lightweight version of a PANA client (PaC) for Contiki OS [60] (PANATIKI) by adapting PANA for constrained devices. It implies removing part of the PaC state machine to make it suitable for constrained devices. Although PANATIKI does not make any modification to the standard, it does not implement some parts of the standard PaC state machine. In other words, it represents a reduced version of the standard, and a best case for PANA-based solutions. That is why we will make a comparison with PANATIKI to evaluate our proposal against an implementation that is optimized for constrained devices.

It is important to note that (the use of) PANA is part of *Zigbee IP* [23]. In particular, the standard proposes the use of a PANA and EAP-TLS method [61] (based on X.509 certificates) for the EAP authentication. They use EAP in standalone mode without AAA infrastructures. Nevertheless, our CoAP-based EAP lower-layer could be used instead of PANA.

Additionally, the *Institute of Electrical and Electronics Engineers* (IEEE) association in the standard IEEE 802.15.9 [62] proposes a transport method for key management protocol (KMP) datagrams that will make use of existing KMPs with the IEEE 802.15.4 and .7. Guidelines will be provided regarding the use of KMPs like HIP, IKEv2, IEEE 802.1X and PANA. On another note, the *European Telecommunications Standards Institute* ETSI [63] defines the support for Generic Bootstrapping Architecture (GBA) and adopts PANA as an option.

Thus, as observed, the previous set of solutions that consider EAP and, possibly, AAA offer a range of alternatives. The references proposing a concrete solution focus on the use of PANA, such as the case of PANATIKI. However, we argue that a lighter protocol, CoAP, is still possible to provide a lightweight bootstrapping process.

It is worth mentioning there is also another set of solutions [64,65,66,67,68,69] that show the need for a bootstrapping process but they do not consider EAP or AAA infrastructures as part of their solution. In this sense, they do not support federated authentication and authorization, so limiting the deployment to small or medium scale scenarios. For example, Garcia-Morchon *et al.* [64] propose two different architectures providing secure network access, key management and secure communications. The first solution uses a variant of HIP-DEX based on pre-shared keys and the second solution uses DTLS. For secure network access, a pre-shared key is assumed (*i.e.*, manually configured by the administrator) between a domain manager and the constrained device. This key is used in HIP-DEX or DTLS in order to authenticate with the domain manager and gain access to the network.

Alternatively, Bergmann *et al.* [65] propose a bootstrapping solution with CoAP. First, the starting node discovers another node that can assist in the bootstrapping. This helping node serves to distribute a temporary secret and establish an association based on DTLS with the pre-shared key (DTLS-PSK). This security association is used to obtain a final session key. With this final session key a new DTLS session can be established. The critical part of the solution lies in the fact that the temporary shared secret is sent in the clear with no protection. Again, the issue with this approach is that the envisioned scenario is only valid for small scale deployments, such as home automation systems.

Korhonen [66] adapts the 3GPP’s *Generic Bootstrapping Architecture* (GBA) [47] to fit in IoT networks. The solution maps the protocols used in GBA, such HTTP and TLS, to its IoT counterparts, CoAP and DTLS, respectively. To simplify the bootstrapping process, a lightweight GBA bootstrapping architecture is proposed. This architecture assumes some pre-configuration of symmetric keys and renders the AAA server unnecessary. As a consequence, the solution is devoid of the AAA’s architecture scalability. Moreover, it does not use EAP and it only allows one authentication mechanism, relegating the usability to small/medium scale deployments, such as residential networks.

The *Open Mobile Alliance* (OMA) [67] defines a protocol for managing IoT infrastructures called *OMA Lightweight Machine to Machine* (OMA LWM2M). Among other aspects, bootstrapping is defined using CoAP and DTLS. The architecture consists of three entities, *LWM2M Client*, *LWM2M Server* and *Bootstrap Server*. It is specified that the Client and the Server share credentials with the Bootstrap Server in order to authenticate, but it is not specified how the credentials are configured.

The *IPSO Alliance* [68] defines the use of CoAP as application protocol, using DTLS to protect the devices with sensible resources such as actuators unless other underlying security mechanism are used. No further considerations are added to the security and bootstrapping landscape.

Finally, the *World Wide Web Consortium* W3C [69] considers the case of security on the smart object bootstrapping outside its scope, proposing the adoption of other security implementations such as the *ARM Trust Zone* [70], although there is a proposal aimed at using of GBA [71].

Thus, these solutions do not support large scale deployments, specially for the lack of identity federation support. However, we envisage the necessity of scalable systems where smart objects from different vendors and organizations can interoperate in large scale scenarios, for example, smart cities.

## 3. The Bootstrapping Service: CoAP-EAP

Our bootstrapping service for IoT rests on three main technologies: CoAP, EAP and AAA. To make this service possible, we have designed a new EAP lower-layer based on CoAP, so it is used to steer an EAP authentication between the smart object and the controller. In turn, the controller interacts with a backend AAA infrastructure to complete the authentication and authorization steps required in the bootstrapping.

As a consequence of the *bootstrapping phase*, fresh cryptographic material is generated and shared between the smart object and the controller to dynamically establish a security association between them. Thus, the smart object will automatically join the controller’s security domain, and the controller will become a trusted third party for the smart object. In this work, we present two examples to build this security association (although other options could be considered in the future): either by integrity protecting (encryption will be considered in the future) CoAP messages at application level with a new CoAP option, named *AUTH* (*AUTH-based protection*), or by establishing a DTLS security association (*DTLS-based protection*).

After the bootstrapping, during the *post-bootstrapping phase*, the smart object is able to access other services in the security domain. These services can be provided by other smart objects or entities, such as a border router to access the Internet service. It is worth noting that our solution offers the framework and the cryptographic material to be used in the post-bootstrapping, although the operation in this phase is considered as future work. For example, after the bootstrapping, the controller may act as a key distribution center as is specified in [72] or as an authorization server, as specified in [73].

By defining a bootstrapping service with these technologies, we propose a solution with the following features:

- *Constrained and low-overhead*. CoAP is designed for communications among smart objects in constrained networks. Moreover, we assume that the smart object already ships a CoAP implementation to support other services in IoT networks, so we can re-use the source code for the bootstrapping service.
- *Interoperability*. The solution is based on three well-known standards, which promotes interoperability and easy deployment. The influence that CoAP has on constrained devices and their use in IoT environments as an application protocol for smart object management benefits interoperability.
- *Security and well-known key distribution and management*. The use of EAP and its associated key management process and the guidelines for AAA key management defined in [13] provides a mature framework for key management.
- *Flexibility*. The use of EAP and AAA provides flexibility in the authentication and authorization processes, so they can be easily adapted to the needs of IoT networks.
- *Scalability and large scale deployment*. AAA framework is already deployed to support millions of users nowadays, for example in 3G networks.
- *Federation support*. AAA provides federated authentication and authorization by design.

The constrained devices that will be able to benefit from this solution will be devices of classes 1 and 2 as described in [37]. Class 0 devices are not considered a target of this solution because of their constraints in memory and processing capabilities as they are not expected to have the resources required to communicate directly with the Internet in a secure manner.

Below, we describe the details of the architecture of our bootstrapping service and how the entities involved are mapped to the *EAP Key Management Framework* (KMF) and the AAA framework we have described in Section 2.

### 3.1. CoAP as EAP Lower-Layer

One of the first questions that we should answer is the suitability of CoAP as EAP lower-layer. In particular, the requirements for a correct EAP lower-layer are specified in [12]. We can affirm that CoAP can be used as EAP lower-layer by contrasting its capabilities with these requirements:

*Unreliable transport*. Although EAP does not assume that lower layers are reliable, CoAP provides reliability by means of Confirmable messages. This implies that retransmission timers at EAP level can be stopped for simplification, as recommend in [12]; *Lower layer error detection*. EAP assumes the lower layer has mechanism of error detection. CoAP is performed on top of UDP which already provides a checksum over the whole payload, where CoAP is transported; *Lower layer security*. EAP does not require lower layers to provide security services. CoAP exchanges can be performed without security; *Minimum MTU*. EAP requires a EAP lower-layer with a MTU size of 1020 octets or greater. CoAP assumes an upper bound value of 1024 octets in the payload, where EAP will be transported; *Possible duplication*. EAP does not require handling duplication of packets. Even so, CoAP provides a Message-ID for deduplication, which does not harm the EAP authentication process; *Ordering guarantees*. EAP requires the lower-layer to preserve the ordering. CoAP allows us to preserve this ordering by using the Message-ID values. Our bootstrapping service uses this field for that purpose, as described in Section 3.3.

As observed, CoAP is able to cover each of the requirements and our bootstrapping service can safely rely on CoAP as a transport for EAP.

### 3.2. Proposed Architecture

To handle the EAP authentication involved during the bootstrapping service, we have designed a new EAP lower-layer based on CoAP. Basically the idea consists of transporting EAP packets in the payload of the CoAP messages involved during the service execution. In general, the Smart Object performs the *CoAP server role* and the Controller the *CoAP client role*. This decision is further explained in Section 3.6.1. To save system resources, it is assumed that the Smart Object will have only a single ongoing bootstrapping exchange and will not process simultaneous EAP authentications in parallel with the same Controller.

**Figure 3:**
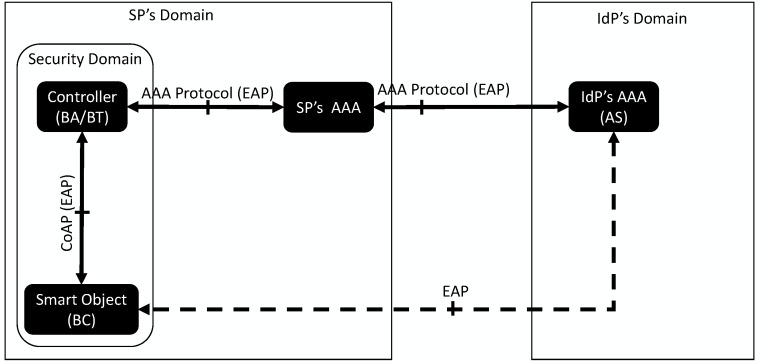
The constrained application protocol (CoAP)-EAP architecture.

Figure 3 shows the architecture of our bootstrapping service using CoAP as a transport for EAP packets between the Smart Object and the Controller. We assume that a Controller manages a security domain and, therefore, the bootstrapping process. In this way, any smart object or entity wanting to join the security domain will have to engage with the Controller.

The Smart Object acts as *end user* in the AAA framework, and it will act as EAP peer and perform the client-side of a particular EAP method. The Controller acts as the EAP authenticator for the bootstrapping service and acts as the EAP authenticator (typically in pass-through mode for big scale deployments) and ships a AAA client (RADIUS or Diameter) to interact with the backend AAA infrastructure. The EAP server is typically placed in the IdP’s AAA server where the Smart Object is registered. Several intermediate AAA proxies can be placed between the Controller and the IdP’s AAA server, especially in federated environments (although it will depend on the specific deployment) [20].

After finishing the bootstrapping service, the Controller becomes a trusted third party in the security domain for the Smart Object so that it can interact in a secure fashion with the rest of the entities within the domain.

### 3.3. General Operation Flow

In order to run the bootstrapping service, it is necessary to define a *Uniform Resource Identifier* (URI) so that any endpoint can refer to the service using that value. In particular we defined the URI as */boot* for our bootstrapping service.

In this manner, when the Smart Object, acting as CoAP client, wants to start the bootstrapping service, it sends a Confirmable *POST /boot* request to the Controller, which acts as CoAP server in this first exchange (step **1**). Typically, the Controller will answer back with a response message (step **1’**) saying the service is available (CoAP Response Code *2.04*). However, it may omit it and proceed with the following exchange to save this message in the link. Then, the Controller, as CoAP client for the rest of the exchange, immediately sends a Confirmable *POST /boot* request to the Smart Object as CoAP server from this point on (step **2**). The Smart Object indicates the creation of a resource for the bootstrapping service to the Controller. The message carries a nonce (*nonce-c*), which will be used for the generation of fresh cryptographic material after the bootstrapping execution; and a Token with a value chosen randomly. This value is used as the session identifier and kept during the whole authentication. The smart object assigns an identifier to a resource (value 5 in the example) and answers with an ACK that carries a piggybacked response with a new nonce (*nonce-s*), also used for the same purpose than *nonce-c*, and the Token (step **3**).

The *Message-ID* (MID) values in the requests sent by the Controller are generated *randomly*, as suggested in the CoAP standard. The Controller selects a new Message-ID value each time a new request is sent to the Smart Object, until the bootstrapping service finishes. Moreover, the Controller stores the last Message-ID sent until correctly receiving the corresponding ACK. The Smart Object keeps track of the last received Message-ID to identify retransmissions, and the previous Message-IDs during the current bootstrapping to identify old messages. In general, a request is considered valid in terms of the Message-ID if either this value matches the last value received, which means a retransmission of the last response is required, or the arrival of a new Message-ID, which therefore represents a new message. If these rules do not apply (*i.e.*, an old Message-ID has been received), the Smart Object silently discards the request. This is possible because the bootstrapping service is designed as lockstep: the Controller will not send a new request until receiving the corresponding response. When the current bootstrapping exchange finishes successfully, the smart object can free the tracked Message-IDs, except for the last received Message-ID at the end of the bootstrapping, just in case a retransmission is required.

**Figure 4:**
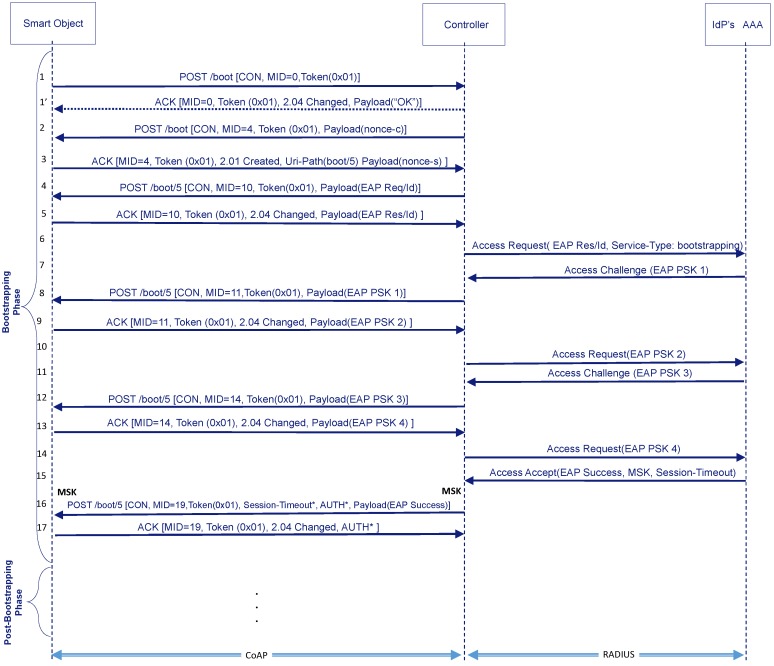
CoAP-EAP bootstrapping service flow (using EAP-pre-shared key (PSK) as example of EAP method).

After this initial handshake, the EAP authentication starts. Figure 4 shows an example of exchange using the EAP-PSK method and pass-through mode (IdP’s AAA server intervenes in the EAP authentication). Nevertheless, the number of messages will depend on the EAP method used. The Controller will use the POST method to send EAP requests to the resource created in the Smart Object. Then, the Smart Object sends an ACK with a piggybacked response to carry the EAP responses to the Controller (steps **4**–**16**). This corresponds with *phases 1a and 1b* in the EAP KMF (RFC 5247 [14]).

Specifically, the Controller first requests the Smart Object’s identity (*EAP Req/Id*) (e.g., *smartobject@domain.net*) (step **4**). Then it sends its identity (*EAP Res/Id*) in an ACK message (step **5**). The Controller uses the information in the identity to route the EAP Response/Id message to the IdP’s AAA. Without loss of generality, our example is based on RADIUS as AAA protocol. In Figure 4 we can observe the RADIUS *Access-Request* message containing the EAP Res/Id (step **6**). To inform the IdP’s AAA that this EAP authentication is for a bootstrapping purpose, the Controller includes the *Service-Type* attribute with a new value: *Bootstrapping*. This is required so that the IdP’s AAA can apply authorization decisions adapted to the bootstrapping service.

Based on this information, the IdP’s AAA server selects the proper EAP method for authentication. In our example, this will be the EAP-PSK method, which implies 4 messages (steps **7**–**14**). EAP requests of EAP-PSK are transported to the Controller from the IdP’s AAA server by using *Access-Challenge* messages and responses are received from the Controller by means of *Access-Request*.

At the end of the message exchanges, if everything has gone as expected, the Controller receives cryptographic material (*i.e.*, MSK) from the IdP’s AAA server along with the *EAP Success* message in a *Access-Accept* message. In turn, the Smart Object will be able to generate the same keying material as defined by the EAP KMF specification. In addition to this information, the Controller may receive authorization information from the AAA infrastructure, for example, the session lifetime (*Session-Timeout* attribute in RADIUS) related with this bootstrapping process. Part of this information may be passed to the Smart Object through, for example, a generic CoAP option named *Authorization Option* (step **16**). All this information is stored in the so-called *bootstrapping state* (see Section 3.5).

At this point, the Smart Object and the Controller share cryptographic material (MSK) to establish a security association enabling the signaling to be protected between both entities for further service requests. This corresponds to *phase 2a* in the EAP KMF [14]. In this paper, we show two examples of usage of this keying material, though other alternatives may be considered in the future: first, the integrity protection at application level of CoAP messages by means of a new AUTH option; second, the establishment of a DTLS security association. In the following sections, we present some details about these alternatives, describe how to manage the bootstrapping state and provide additional considerations.

### 3.4. Bootstrapping Security Associations

#### 3.4.1. Key Hierarchy Design

The first step to protect services and messages after the bootstrapping is to design a key hierarchy. If AUTH-based protection is used (see Section 3.4.2) a new key named COAP_PSK is derived. In contrast, if DTLS is used, a DTLS_PSK is derived (see Section 3.4.3).

Additionally, we have considered the derivation of an *Application-Specific Root Key* (ASRK), which is used as a root of an additional key hierarchy. By using the ASRK, the Controller can acts as key distribution center for the recently bootstrapped Smart Object. For example, this key material will allow the Smart Object, if properly authorized, to securely access to the services offered by the Controller, other bootstrapped Smart Objects or entities within the security domain, which also have a security association with the Controller (the trusted third party for all of them). How this key is used will depend on the interactions expected in the security domain and the services accessed during post-bootstrapping. In fact, it could be used to distribute key material to layer 2 or layer 3 security. In any case, it is left out of this work and will be covered as part of future work.

#### 3.4.2. CoAP Message Protection at Application Level: AUTH Option

Once the Controller has received the MSK from the AAA server, it can derive the COAP_PSK. This key is used to generate the values for the new defined AUTH option, which contains a *Message Authentication Code* (MAC) for integrity protection over the entire CoAP message. The reason for using this option is that it has been proved that running a DTLS handshake can be really costly in terms of time in IEEE 802.15.4 networks [74]. Thus, certain applications may prefer not to perform the DTLS handshake. In fact, other types of (non-IEEE 802.15.4) networks considered in IoT, generically referred to as LPWA (or LPWAN) for *low power wide area (network)* [75] are generally low power and low-throughput [76] and, therefore, saving bits in the link is a benefit.

**Figure 5:**
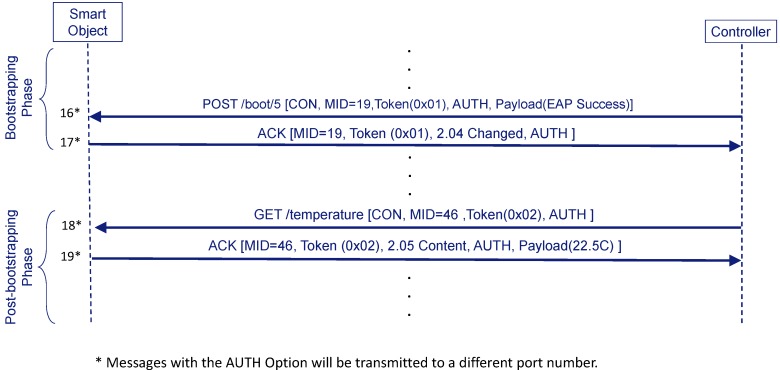
AUTH-based protection example.

In Figure 5 we can see an example of message protection with the AUTH Option.The first time this option appears is during the bootstrapping phase in the message that conveys the EAP Success (step **16’**). The corresponding ACK message (step **17’**) from the Smart Object, which closes the bootstrapping phase, also includes an AUTH option. By verifying the MAC, one endpoint can know whether the other endpoint owns the COAP_PSK.

Since AUTH option is a new type of protection, a new port (to be assigned by IANA) and a new URI scheme identifier (e.g., “coapa”) should be allocated. Thus, this last exchange will go through this new port. From this point, any message related with the bootstrapping service (e.g., to remove the bootstrapping state as explained in Section 3.5) will include the AUTH option.

Any other service (e.g., to obtain the temperature from the smart object), implying the communication between the Smart Object and the Controller, can also use the AUTH option (steps **18’** and **19’**). Nevertheless, this does not preclude the use of DTLS, as we will see in Section 3.4.3 with the derivation of the DTLS_PSK. In fact, the use of either DTLS or AUTH will depend on the service URI, “coaps” or “coapa”, respectively. The use of different URIs to indicate the protection of the exchange are exclusive. The reason is that it is not useful to provide integrity with the AUTH option and ciphering with DTLS, since DTLS already offers the possibility of using integrity and ciphering. Hence, the use of the AUTH option is used when a very simple and less taxing approach is needed.

COAP_PSK is a 16-byte length key which is computed using AES-CMAC-PRF-128 [77] as *Key Derivation Function* (KDF), which, in turn, uses AES-CMAC-128 [78]. Both primitives use AES-128 [79] as building block since it is widely used in constrained devices.

(1) $$COAP\_PSK=KDF(MSK,\prime \prime IETF\_COAP\_PSK^{\prime \prime }||nonce-c||nonce-s,64,length)$$

where

The *AES-CMAC-PRF-128* is defined in [77]. This function uses AES-CMAC-128 as a building block; The *MSK* is exported by the EAP method; “IETF_COAP_PSK” is the ASCII code representation of the non-NULL terminated string (excluding the double quotes around it). This value is concatenated with the value of the nonces exchanged; *64* is the length of the MSK; *length* is the length of the label “IETF_COAP_PSK” (13 bytes) plus the two nonces; *nonce-c* is a random value sent from the Controller to the Smart Object; *nonce-s* is a random value sent from the Smart Object to the Controller.

To calculate the MAC value, an endpoint inserts the AUTH option and sets its value to 16 bytes with zero. Then, it applies the function AES-CMAC-128 to generate the AUTH Option value over the entire message as follows:

(2) AUTHOptionvalue=AES-CMAC-128(COAP_PSK,message,length)

where

*COAP_PSK* is the key derived in Equation (1); *message* is the CoAP message including the AUTH option filled with zeros; *length* is the length of the CoAP message including the AUTH option.

It is worth mentioning that while we propose the use of an AUTH Option just as an example to show the protection of CoAP messages at application level, there are additional works, such as [80], that define additional CoAP Options to cope with protection at application level. In parallel, Object Security for CoAP (OSCOAP) has been considered [81] to protect CoAP messages with *CBOR Object Signing and Encryption* (COSE) [82] objects.

#### 3.4.3. CoAP Message Protection with DTLS

**Figure 6:**
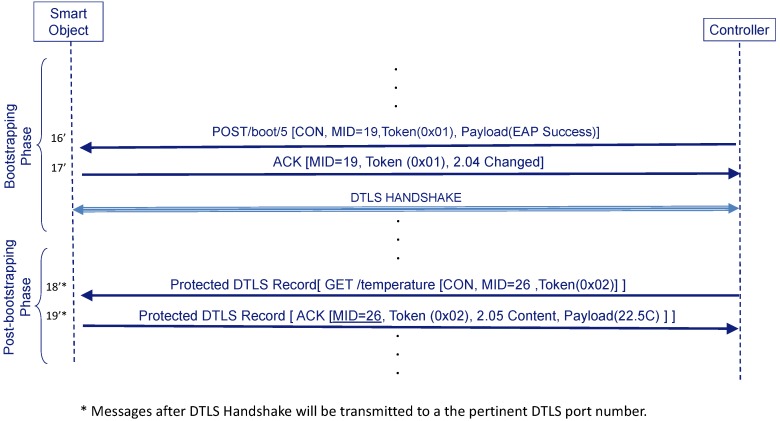
Protecting post-boostrapping with datagram transport layer security (DTLS).

DTLS is the standard protocol used to protect CoAP messages by default. If the Controller wants to use DTLS for protecting messages it will not send the AUTH option (see Figure 6) (step **16”**). In this case, it derives a DTLS_PSK from the MSK (the Smart Object will do the same) and starts a DTLS negotiation over port 5684. Thus, the Smart Object and the Controller do not consider the bootstrapping to be complete until the DTLS handshake finishes successfully.

Now, messages related with the bootstrapping service that may be exchanged from this point (*i.e.*, Confirmable DELETE) are protected by DTLS. Any other service can still use DTLS (steps **18’** and **19’**) if the URI scheme for this service contains “coaps” (or even decide to use AUTH option deriving the COAP_PSK if the URI scheme contains “coapa”).

The DTLS_PSK will also have 16 byte length and will be derived as follows:

(3) $$DTLS\_PSK=KDF(MSK,\prime \prime IETF\_DTLS\_PSK^{\prime \prime }||nonce-c||nonce-s,64,length)$$

where

*MSK* is exported by the EAP method; *“IETF_DTLS_PSK”* is the ASCII code representation of the non-NULL terminated string (excluding the double quotes around it); *64* is the length of the MSK; *length* is the length of the label “IETF_DTLS_PSK” (13 bytes) plus the two nonces; *nonce-c* is a random value sent from the Controller to the Smart Object; *nonce-s* is a random value sent from the Smart Object to the Controller.

As mentioned in [83], a PSK identity is needed. We consider the use of the Token value chosen during the EAP authentication as PSK identity.

### 3.5. Bootstrapping State Definition and Management

The *bootstrapping state* is a set of parameters resulting from the bootstrapping process described in Section 3.3. This state is maintained by the Smart Object and the Controller. The bootstrapping state comprises the following values: both entities’ IPv6 addresses; authorization information related with the bootstrapping service that came form the AAA infrastructure, in particular, the supported cryptosuite, capabilities (e.g., AUTH-based or DTLS-based protection) of the Smart Object and the lifetime associated to the state; list of keys exported and derived from the bootstrapping procedure (COAP_PSK, DTLS_PSK and ASRK) and a resource identifier generated in the Smart Object after receiving a Confirmable POST /boot request.

In general, if the lifetime of the bootstrapping state expires at both endpoints it will be automatically removed, along with the associated resource. Nevertheless, the Smart Object or the Controller can explicitly signal a desire to remove the bootstrapping state.

**Figure 7:**
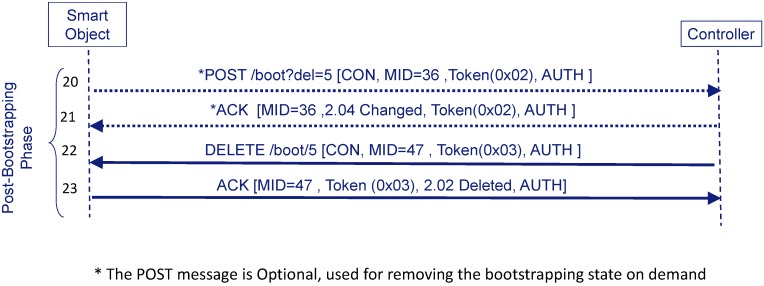
Example of deleting bootstrapping state (with AUTH-based protection).

On the one hand, the Smart Object may also request the Controller to abandon the security domain and, thus, to delete bootstrapping state. This is useful because the Smart Object may desire to notify the Controller that it should stop sending accounting information to the AAA infrastructure since, for example, it is leaving the security domain. In a similar way as the Smart Object signals that it wants to start a bootstrapping by sending a Confirmable POST message to the URI of the bootstrapping service (*POST /boot*), the Smart Object can signal the Controller its desire to leave the security domain by the following URI-Path: */boot?del=X*. The part */boot* indicates that the Smart Object requires the Controller to perform some action related to the bootstrapping. This action is defined by the part *?del=X*, which signals to the Controller that the Smart Object desires to leave the security domain, where *X* is the resource identifier to be erased. For example, Figure 7 shows the Smart Object sends a protected *Confirmable POST /boot?del=5* (step **20**) to trigger the Controller to remove resource identifier 5. When the Controller receives this request, it looks for an existing bootstrapping state that matches the identifier. If the Controller has the bootstrapping state, it sends a Confirmable DELETE request (step **21**) to the Smart Object to erase the resource with the assigned identifier (e.g., *“boot/5”*). Typically, the Controller can also send the response associated to the POST but the Smart Object considers that receiving the DELETE directly is sufficient. In fact, upon receiving the Confirmable DELETE request, the Smart Object sends a protected ACK with piggy-backed response (step **22**) with a response code to confirm the bootstrapping state is going to be removed (*2.02*). If the Controller already removed the state (e.g., it already expired), it sends an unprotected (there is no bootstrapping state) ACK message with code *4.04 (Not Found)*. The Smart Object may not trust this unprotected message and insist on sending POST (e.g., 3 times) to see if it receives a protected Confirmable DELETE request. If the Smart Object does not finally receive this request, it may consider that the Controller has already removed the state. On the other hand, if the Controller wants to delete the bootstrapping state, it only has to start the Confirmable DELETE request/ACK exchange.

Finally, the Smart Object may want to renew the bootstrapping state (refreshing cryptographic material, lifetime, *etc.*) before it expires. This means a new EAP authentication, as we have described in Section 3.3. This new bootstrapping exchange needs to finish before the current bootstrapping state expires. If the new bootstrapping process finishes successfully, the current bootstrapping state is replaced with the new one.

### 3.6. Additional Considerations

#### 3.6.1. CoAP Role Selection

With the exception of the *POST /boot* message sent by the Smart Object to notify the Controller the beginning of a bootstrapping process, or the *POST /boot?del=X* to trigger the removal of resource *X*, the rest of exchanges assume that the CoAP server is the constrained Smart Object and the CoAP client is the Controller. The main reason for this role choice is, as suggested by [84], to simplify the Smart Object implementation, assuming this will be the most constrained entity. Additionally, in EAP, the authenticator sends EAP requests from the peer, which returns an EAP response. The authenticator carries the burden of retransmissions including additional states for this purpose in the EAP state machine [85]. Similarly, in CoAP, the CoAP client sends CoAP requests, and the CoAP server just answers back with a response. With our design choice, a CoAP request carries an EAP request and a CoAP response transports an EAP response, avoiding the odd case where EAP requests go into CoAP responses and vice-versa, which complicates the overall design.

#### 3.6.2. Discovering the Controller

One aspect that has not been discussed until now is how the Smart Object discovers the Controller. Or, in other words, how the Smart Object knows which entity supports the bootstrapping service in a particular security domain. Actually, this process lies outside the bootstrapping service, since there are already mechanisms for that process in CoAP. For example, the CoRE Resource Directory [86] provides the framework to discover services.

In particular, it describes an entity called *Resource Directory*, which can register the bootstrapping service (*rt = ”bootstrapping”*). The access to this information should be public and not protected since it will be the first service to be used for Smart Objects that want to join the security domain. The Resource Discover will return a “coap” URI schema (e.g., coap://[IP-Controller]/boot) for our bootstrapping service, meaning the initial exchanges goes to the unprotected CoAP port.

#### 3.6.3. Trusting the Controller

One question that may remain is how the Smart Object can trust the Controller. Basically this is explained and discussed in the EAP KMF (Sections 2.3 and 3.1 Part b) [14]. In short, the trust is based on the fact that the controller receives and uses (proof of possession) the MSK (in reality a key derived from the MSK such as COAP_PSK or DTLS_PSK) to establish a security association after a successful authentication. Since the IdP’s AAA server provides this MSK to the Controller and the Smart Object trusts the IdP’s AAA server, if the AUTH option is verified correctly it means the controller obtained the MSK from a trusted entity (the IdP’s AAA server) and derived the COAP_PSK. Alternatively, if the DTLS handshake is used and finishes correctly, it means the Controller was able to derive the DTLS_PSK from the MSK.

Additionally, the Controller needs to be trusted by the AAA infrastructure. This is typically done by the SP administrator configuring the Controller with a credential (e.g., a shared secret) that can be verified by the SP’s AAA server. Moreover, the SP’s AAA server is configured to register the Controller as a trusted entity. In the same way, SP’s AAA server is trusted by the IdP’s AAA server. Thus, the Controller is trusted by the AAA infrastructure for a principle of trust transitivity, which is the common model in AAA infrastructures [11].

#### 3.6.4. Authorization Aspects

Authorization is also an important part of the bootstrapping process. The authorization information related to the bootstrapping service (*bootstrapping authorization information*) determines, for example, whether the Smart Object can join the security domain and under what conditions. Since our bootstrapping solution is based on AAA framework, the authorization information is carried to the Controller in the form of AAA attributes. RADIUS and Diameter define a myriad of attributes (e.g., NAS-Filter-Rules, Framed-MTU, Session-Timeout, *etc.*) to carry this information. For more fine-grained authorization, [87] has recently specified how to transport SAML in RADIUS attributes, though more constrained authorization information (in term of message size) could be expressed in JSON [88] or CBOR [89] format. However nothing has yet been defined in this regard.

We consider that the set of attributes already defined in RADIUS or Diameter could be a good starting point for a basic and correct authorization of the bootstrapping service. In particular, we have paid attention to the lifetime associated to the bootstrapping state or the service type. Nevertheless, if other types of information are required (for example, certain domains may require a hardware profile of the Smart Object to take an authorization decision regarding the Smart Object, even though it is successfully authenticated), this may require the definition of new IoT-based AAA extensions, such as [90]. Nevertheless, the AAA framework is prepared to be easily extended so giving the required flexibility to provide any application-specific authorization information needed for IoT applications.

Additional authorization information may be required *after* the bootstrapping for the operation in the security domain. For example, this is required for accessing services provided by other Smart Objects, the Controller, border routers, *etc.*, in the security domain. This information is also application specific and considered as part of future research. Nevertheless, the IETF ACE WG is investigating solutions for this post-bootstrapping authorization process.

Finally, how the Controller will handle any authorization information will depend on the local security policies and the activity of the Smart Object in the security domain. Additionally, if some authorization related information needs to be carried to the Smart Object we will use our CoAP-based EAP lower-layer for the transport. The general framework to support this is using CoAP options containing the required information. For example, an *Authorization* Option may be included in the CoAP message to notify the Smart Object with the bootstrapping authorization lifetime value, if this value is different from the default, which is 8 hours following the hint in [14]. Knowing the session lifetime will allow the Smart Object to arrange a new bootstrapping before the existing bootstrapping state expires.

#### 3.6.5. Cryptographic Suite and Protection Selection

In order to simplify the bootstrapping service, we assume a default cryptographic suite based on AES algorithm. We assume AES-CMAC-PRF-128 as the default KDF (see Section 3.4.3); AES-CMAC-128 to generate the new AUTH option (Section 3.4.2); and, when encryption and integrity protection are required at CoAP application level, we assume AES-CCM [91]. In any case, some sort of negotiation is important to support cryptographic agility [92].

In our model, the controller is the entity which decides the cryptographic suite that must be used in the security domain. Thus, the information the controller needs to know about the Smart Object is the supported cryptosuite. Since our solution is AAA-centric, the IdP’s AAA, where the smart object is registered, can provide this information to the Controller during the bootstrapping, so that it can finally decide what option to choose. Thus, this information is not sent over the constrained network reducing the CoAP message size of the bootstrapping service. Additionally, the IdP’s AAA can inform if the smart object supports AUTH-based protection, DTLS-based protection or both.

In contrast, the AAA protocol needs to carry this information. However, there are no standard attributes for this. It means defining several new attributes: *Encryption-Type*, *KDF-Type*, *Protection-Type* and *MAC-Type*. These attributes would carry one octet specifying the algorithm (for example, using the value in [93]) for ciphering; the KDF to derive cryptographic material; the function to generate the AUTH option value, and the type of protection supported by the smart object, respectively.

The IdP’s AAA server may include an attribute of this type per each supported algorithm when sending the EAP Success to the Controller. For simplicity, if these attributes are not included, the Controller will assume that the default values are those supported by the smart object and that both AUTH-based protection or DTLS-based are supported.

Finally, the smart object needs to be informed about the decision as well. This can be done in the last POST message containing the EAP Success (step **16**) through a new option *Crypto-Suite* that contains 3 bytes (3-tuple) with: encryption, KDF and MAC algorithms. Nevertheless, to avoid increasing the message size, if this option is not included it is assumed that the default values will be the chosen ones. The use of AUTH-based or DTLS-based protection is determined because the Smart Object sees a message over the DTLS port and protected with DTLS or a message with AUTH option over the yet-to-be defined port for AUTH-based protection.

#### 3.6.6. Other Security Considerations

On the one hand, our bootstrapping service makes use of EAP and AAA and associated key management. In terms of security, the solution does not add anything different than existing deployments of EAP and AAA do. In this sense, it follows the *EAP Key Management Framework* [14] and the *Guidance for Authentication, Authorization, and Accounting (AAA) Key Management* [13].

On the other hand, the AES-based cryptosuite selected as default is well-known and already deployed nowadays in IoT devices [94]. It is worth noting that the cryptosuite negotiation is for the operation at bootstrapping service level. In other words, the DTLS negotiation can select a different set of algorithms.

Nevertheless, we should clarify how we avoid any downgrading attack during the cryptosuite selection procedure shown in Section 3.6.5. This attack happens when an attacker removes some of the algorithms to limit the set of valid cryptographic algorithms to those more favorable to the attacker. We assume that AAA infrastructure is trusted; therefore the path between the Smart Object and the Controller is where the attack may happen. Our mechanism to detect this attack is simple. If protection at level application is enabled (AUTH option) the last POST message and corresponding ACK (Figure 4—steps **16** and **17**) are integrity protected so any modification (removal or modification of the algorithms specified in the POST message) will be detected by both entities. On the contrary, if DTLS is used, we can observe in Figure 6 that the last POST message and corresponding ACK are not protected. This is why the Smart object and the Controller must end the DTLS negotiation before considering the bootstrapping complete. In reality, the KDF is the only function which needs to be agreed in this case, since DTLS already performs a cryptosuite negotiation. Thus, if an attacker has modified the KDF algorithm, the DTLS_KEY derived by the Controller and the Smart object will be different and DTLS will fail, so that the attack is detected.

## 4. Experimental Results

In this section we present the testbed we have defined to evaluate the performance of the CoAP-EAP implementation done for this purpose. To complete the evaluation and analysis we compare CoAP-EAP with PANA-based solutions, which have a similar purpose but the EAP lower-layer is based on PANA. As a representative of PANA-based solutions, we have chosen PANATIKI since it can be considered as a best case for these type of solutions. The reason is that PANATIKI is a design and implementation of PANA optimized for constrained devices.

In particular, we have compared CoAP-EAP with AUTH-based protection against PANATIKI. The reason is that, after bootstrapping, running DTLS can be considered as the same task in both alternatives, thus not adding value to the comparison. For the performance of DTLS, the interested reader is referred to [74]. By including AUTH option we show the worst case in terms of the operation of our CoAP-based EAP lower-layer (it has to process the AUTH option) against the best case of PANA-based solutions, which is PANATIKI. Even so, there is still some room for improvement, as we will analyze in the next sections.

### 4.1. Experimental Setup

**Figure 8:**
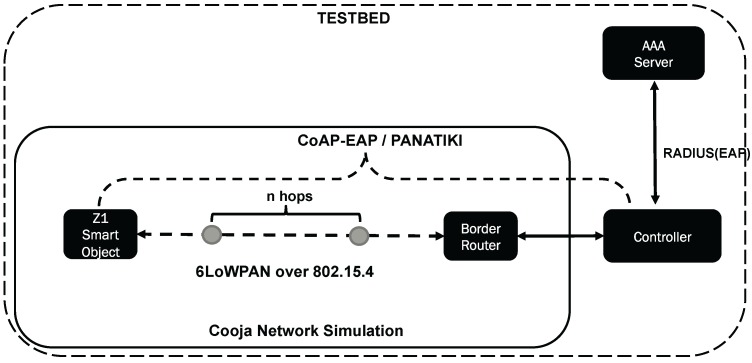
Testbed for the evaluation.

Figure 8 shows the testbed we have prepared for our performance evaluation. For the testbed we use the Cooja Network Simulator with Contiki OS in its version 2.7 [95]. The Smart Objects used for this testbed are the Zolertia Z1 with 92 kB of nominal ROM when compiled with 20-bit architecture support, and 8 kB of RAM. The compiler is *msp430-gcc version 4.7.2*.

There is an entity in Cooja, the *RPL border router*, that enables the communication between the Cooja Network and the outside physical network where the Controller is located. Between the border router and the Smart Object there can be 1 hop (direct link), or other smart objects between the Controller and the Smart Object performing the bootstrapping. The idea of having several hops is to observe the behavior of the bootstrapping time when intermediate Smart Objects are acting as IP-forwarders.

For the tests, we used a 4-byte length Token in CoAP-EAP, which provides a 32-bit for the session identifier, as in the case of PANA. Following the recommendations in [96], we have performed the simulations in Cooja with a randomly generated seed to automate the process of running the simulations. We have used the default parameters in Contiki for the MAC layer and RDC. In particular, the parameters for the simulation includes the *contikimac_driver* for RDC and *csma_driver* for CSMA with default values. Due to the length of the PANA messages, we have had to set the parameter UIP_CONF_BUFFER_SIZE to 250 in the Contiki OS so that PANATIKI works correctly (otherwise no PANA authentication was completed). The same parameter is kept in CoAP-EAP for fair comparison.

In term of the software packages, on the one hand, we have used the PANA agent (PAA) implementation of OpenPANA [97] for the Controller in PANATIKI [98]. With the purpose of making a fair comparison, we have implemented the Controller-side of our CoAP-EAP bootstrapping service using the same PAA implementation but replacing PANA with our CoAP-based EAP lower-layer source code. To develop this new EAP lower-layer, we used *cantcoap* [99] as CoAP library. Although we tested several CoAP libraries: libcoap [100], erbium [101] and cantcoap, we decided to go for cantcoap for its simplicity, which gave us greater control over the implementation.

On the other hand, PANATIKI is used to implement the PANA client (PaC) in the smart objects for PANA-based solutions. In CoAP-EAP, we have transformed *cantcoap* from C++ to C version for the compilation in Contiki OS. The same EAP peer state machine of PANATIKI is used in CoAP-EAP. Finally, we have used freeradius [102] version 2.0.2 for the role of AAA Server. The Cooja Simulator, Controller and the AAA Server run in a computer with the specifications shown in Table 1.

For CoAP-EAP and PANATIKI, the EAP method used to obtain experimental results is EAP-PSK due to its lightweight nature. The EAP-PSK keys used are 16 bytes long and the EAP identity is 6 bytes long. The implementation of EAP-PSK is common in both cases. In the EAP peer side, EAP-PSK implementation is the one provided in PANATIKI. On the EAP server side, the EAP-PSK implementation is that in freeradius 2.0.2.

### 4.2. Performance Evaluation

#### 4.2.1. Message Length

In general, the message length may influence the time the Smart Object takes to process it but, more importantly, the time that takes to send and receive it over the network. This aspect gains relevance in lossy networks where, for example, fragmentation becomes a matter of utmost importance [103]. Currently, the IEEE 802.15.4 defines a MTU of 127 bytes.

Table 2 shows the message length (in bytes) for CoAP-EAP and PANATIKI. We show the length of the EAP lower-layer, excluding the length of the EAP message (*LL*) and including the EAP message length (*LL+EAP*). Thus, in the case of CoAP-EAP, *LL* column includes the length of CoAP header (4 bytes), the (variable) list of CoAP options and payload, excepting the length of EAP message itself. As PANATIKI is an implementation of PANA, it follows the standard, so the PANA message length is the same as specified in RFC 5191 [55]. Thus, *LL* column for PANATIKI includes the length of the PANA message excepting the EAP message length. In short, a PANA message includes the PANA header (16 bytes) and a variable length payload. This payload is formed for a list of *Attribute Value Pairs* (AVPs) (e.g., the PANA message containing the EAP success, *PAR(EAP Success)*, includes a list of 5 AVPs). Each AVP has a *Tag-Length-Value* (TLV) format and its length is *8 bytes* plus the length of the content of the AVP. For the specific list AVPs carried in each PANA message during a PANA authentication, the interested reader can refer to [55]. Finally, *LL+EAP* column adds the EAP message length to the values in column *LL* in both cases.

As a consequence, all the messages related with CoAP-EAP have a shorter length in comparison with PANATIKI’s. In fact, it is worth noting that CoAP is designed with a *short fixed-length binary header* and compact binary options [22] and PANA was not designed with the constraints of IoT environments in mind. Overall, there is ≈32% reduction in the number of bytes between both alternatives when looking at LL+EAP. Apart from IEEE 802.15.4 networks, we foresee that this will be also important, for example, in low power wide area networks [75] where sending a single byte is usually very costly. If we pay attention just to the CoAP-based EAP lower-layer that we have designed, since the EAP implementation is common in both approaches, the reduction increases about 50%.

We expect additional impacts of our solution as a consequence of the reduction of message length in networks where the bandwidth is really low. In particular, several discussions are going on in the context of the IETF *IPv6 over the TSCH mode of IEEE 802.15.4e* (6tisch) WG [52] about the use of CoAP as transport of EAP in this type of networks.

Additionally, avoiding fragmentation is important. PANATIKI surpasses this MTU threshold, 127 bytes MTU in IEEE 802.15.4, in three messages (marked with * in Table 2) in contrast with CoAP-EAP, with only one. Although the lower-layer plus EAP message lengths are below 127 bytes, the whole packets including the MAC layer and 6LoWPAN layer, surpasses the MTU. Thus, this is a hint to consider a different EAP method, with shorter messages as well.

#### 4.2.2. Bootstrapping Time

We have defined a scenario with different numbers of hops between the Smart Object and the border router. This allow us to evaluate the performance of each protocol in a more realistic scenario than a simple node connected to a border router.

The tests have been performed with different numbers of hops (*n*), from 1 to 7 hops between the Smart Object and the border router. We have observed though that 7-hop case has proved to be a scenario without connectivity between the Border Router and the Smart Object being bootstrapped. Three different packet loss ratios (0, 0.1 and 0.2) are used to evaluate the response of both solutions.

**Figure 9:**
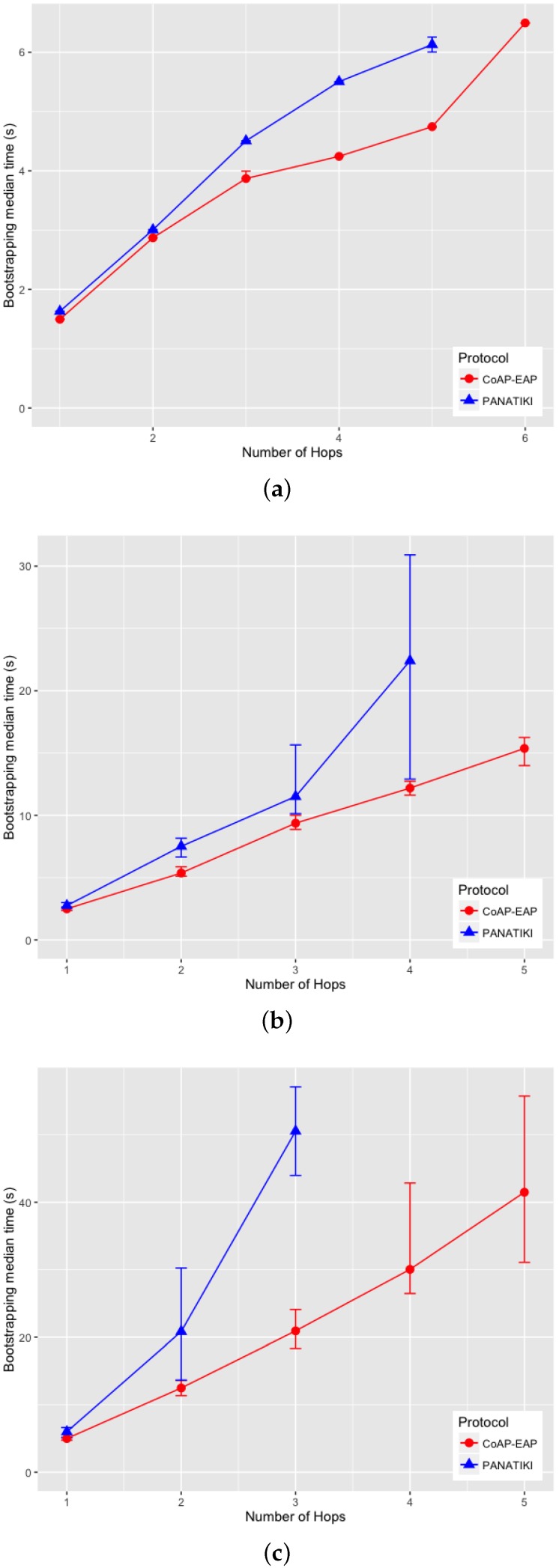
Bootstrapping median Time. (**a**) Bootstrapping Median Time with 0.0 loss ratio; (**b**) Bootstrapping Median Time with 0.1 loss ratio; (**c**) Bootstrapping Median Time with 0.2 loss ratio.

**Figure 10:**
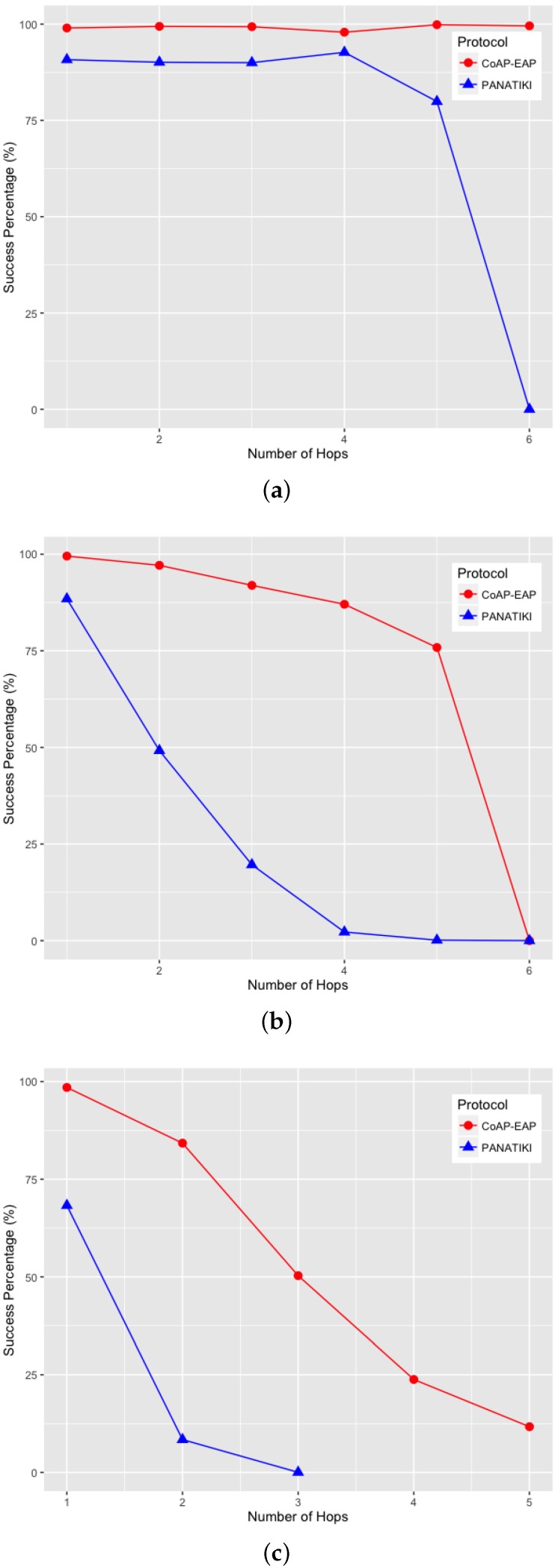
Bootstrapping success percentage. (**a**) Success percentage with 0.0 loss ratio; (**b**) Success percentage with 0.1 loss ratio; (**c**) Success percentage with 0.2 loss ratio.

From the different tests we have gathered the following information: (1) the bootstrapping median time that both alternatives take to complete a bootstrapping for each number of hops and packet loss ratio (Figure 9a–c); and (2) the number of bootstrapping processes that are able to finish (success percentage (%)) in each case (Figure 10a–c).

We can observe in Figure 9a–c that there is a statistically significant difference between the bootstrapping times in PANATIKI and CoAP-EAP. To obtain these values we performed 200 simulations for each value of *n* and for each packet loss ratio.

On analyzing the distribution of the data, we noticed that, a skewness test on the data, returned a value greater than 1. According to Jain [104] a proper index of central tendency is the median rather than the mean, since it provides a more significant description.

In the case of 0 loss ratio and 1 hop, the median values do not differ greatly. In this case CoAP-EAP takes ≈1.5 s and PANATIKI ≈1.6 s. As expected, this difference, partially due to the reduction in message size of our solution (see Section 4.2.1), increases with the number of hops and packet loss ratio since each intermediate smart object has to forward a message and handle fragmentation of these messages. Since PANA has longer messages, PANATIKI provides a longer bootstrapping time than CoAP-EAP. For example, when packet loss is set to 0.2 loss ratio with 1 hop, the median time to accomplish a bootstrapping in CoAP-EAP ≈5 s and ≈6 s in PANATIKI. As expected, this time clearly increases with the increment of number of hops.

Moreover, when the packet loss ratio increases the possibility of completing a bootstrapping decreases. This is implicitly shown in Figure 9a–c. For example, we were not able to complete a bootstrapping process with PANATIKI beyond 5 hops with 0 packet loss ratio; 4 hops when 0.1 packet loss ratio and 3 hops with 0.2 packet loss ratio. However, CoAP-EAP is able to complete the bootstrapping up to 6 hops. This is mainly due to it has a shorter message length and, therefore, it generates less fragmentation. Additionally CoAP has a less aggressive retransmission policy than PANA, which promotes sending less traffic over a constrained network.

To corroborate this, Figure 10a–c show how the success percentage, the bootstrapping processes really finished from those that were started, evolves as the packet loss ratio increases. CoAP-EAP demonstrates a better performance in every packet loss ratio in comparison with PANATIKI. In the worst case scenario with 0.2 packet loss ratio, PANATIKI with 2 hops (1 intermediary node) already has a success of ≈9% which makes the bootstrapping service impractical, whilst CoAP-EAP in the same conditions is able to finish ≈85% of the initiated bootstrapping processes and keeps an acceptable success percentage as the number of hops increases.

#### 4.2.3. Message Processing Time

**Figure 11:**
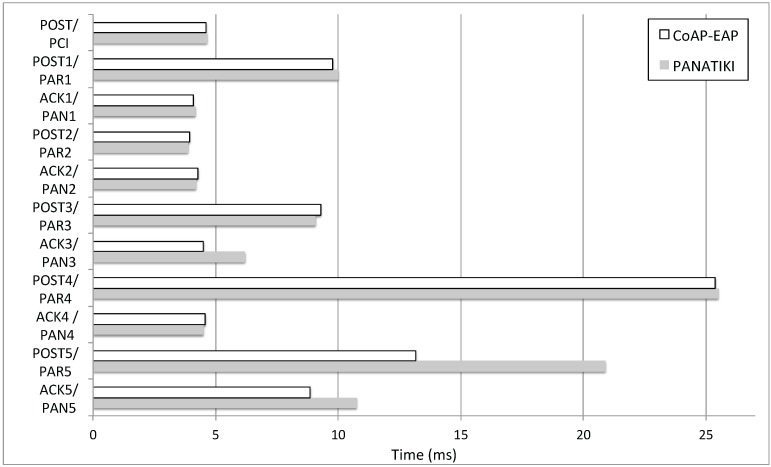
Contiki message processing time.

We have measured the processing time of the messages in the Smart Object (the most constrained device). This time includes the processing of a request and the time that the EAP state machine takes to process the EAP message. The response time measures the time it takes to create the response and send it to the Controller. The results are shown in Figure 11 for each message exchange.

A small advantage can be seen in the last exchange, where the AUTH check and key generation are done in both CoAP-EAP and PANATIKI. This is motivated by two reasons. First, the PANA message is longer than CoAP message and the cryptographic operations are performed over a longer message and, therefore, the cryptographic operations are performed over a longer message. Second, the processing time also includes the PANA_AUTH_KEY generation in PANATIKI and the COAP_PSK generation in the case of COAP-EAP. The COAP_PSK generation is simpler since it involves fewer parameters. For example, PANA includes the first two messages (PAR/PAN) in the key derivation (Section 5.3 in [55]). The reason is that a cryptographic algorithm negotiation process is performed in these two messages and confirmed in the key derivation process. In CoAP-EAP, we do not include this, since our assumption is that a particular KDF will be used and selected by the controller, so no cryptographic negotiation is performed (see Section 3.6.5). In short, the COAP_PSK derivation is simpler than PANA_AUTH_KEY.

With a total average processing time of ≈104 ms for PANATIKI and 93 ms for CoAP-EAP, a ≈11% of reduction in the processing time can be appreciated in CoAP-EAP over PANATIKI in total.

However, if we observe only the EAP lower-layer (the EAP-PSK processing time is common in both alternatives), with a total average processing time of ≈69 ms for PANATIKI and ≈59 ms for CoAP-EAP we can confirm an improvement of ≈15% in CoAP-EAP to process the messages. Although we can observe that CoAP-EAP takes less time to process the messages, this improvement is limited by the EAP method, which has an important weight in the message processing time. In other words, the EAP method implementation has a key impact because it limits the level of improvement. This leads us to conclude that it is important to design very lightweight EAP methods especially adapted for IoT networks.

In any case, if we contrast these values with the effect of fragmentation and message size in the bootstrapping time, we can see that this message processing time is practically negligible.

#### 4.2.4. Memory Footprint

In terms of the memory footprint in the Smart Object, Table 3 shows the size in bytes of several components of the bootstrapping service based con CoAP-EAP or PANATIKI. To obtain these values, we have compiled with memory optimization (-Os compiler option) a set of programs that includes incrementally different modules (EAP, network support, EAP lower-layer) to estimate the size with each new module. Clearly, the EAP state machine and the corresponding modules for enabling network connectivity are also the same.

We observe that the total size of CoAP-EAP solution is slightly bigger than the PANATIKI one. The main reason is the inclusion of cantcoap library, which implements CoAP. Nevertheless we argue that CoAP implementation will surely be present in the majority of the Smart Objects as a common module to be used for other services in the Smart Object, not only for the bootstrapping service. Thus, this library, which adds 4.6 kB to the overall size, is likely to be reused. In fact, it is reasonable to think that PANATIKI will also need to include this library in real deployments to support other CoAP-based services (e.g., Ohba *et al.* [16] requires CoAP also for pulling cryptographic material) giving a total of 102.9 kB + 4.6 kB = 107.5 kB.

Let us illustrate this with an example. Let us assume we have a size of *X* kB available in the Smart Object. Part of that space is occupied by the operating system and the IP/UDP stack for communications. Of course, it is common in both alternatives (62.7 kB + 24.9 kB in our estimation in Table 3). The rest of the available space is to deploy services and applications in the Smart Object. For this, the Smart Object will ship a CoAP implementation (e.g., cantcoap) that will be used for the specific implementation of other services (e.g., a service to obtain temperature measurements). In our case, this CoAP implementation is cantcoap and subtracts 4.6 kB from the free memory available. If we want now to support bootstrapping, we need additional source code. PANATIKI subtracts 5.9 kB but since CoAP-EAP is re-using CoAP implementation it only subtracts 3.8 kB. Thus, CoAP-EAP would save 2.1 kB with respect to PANATIKI in the available space for other services in a more realistic case.

#### 4.2.5. Energy Consumption

**Figure 12:**
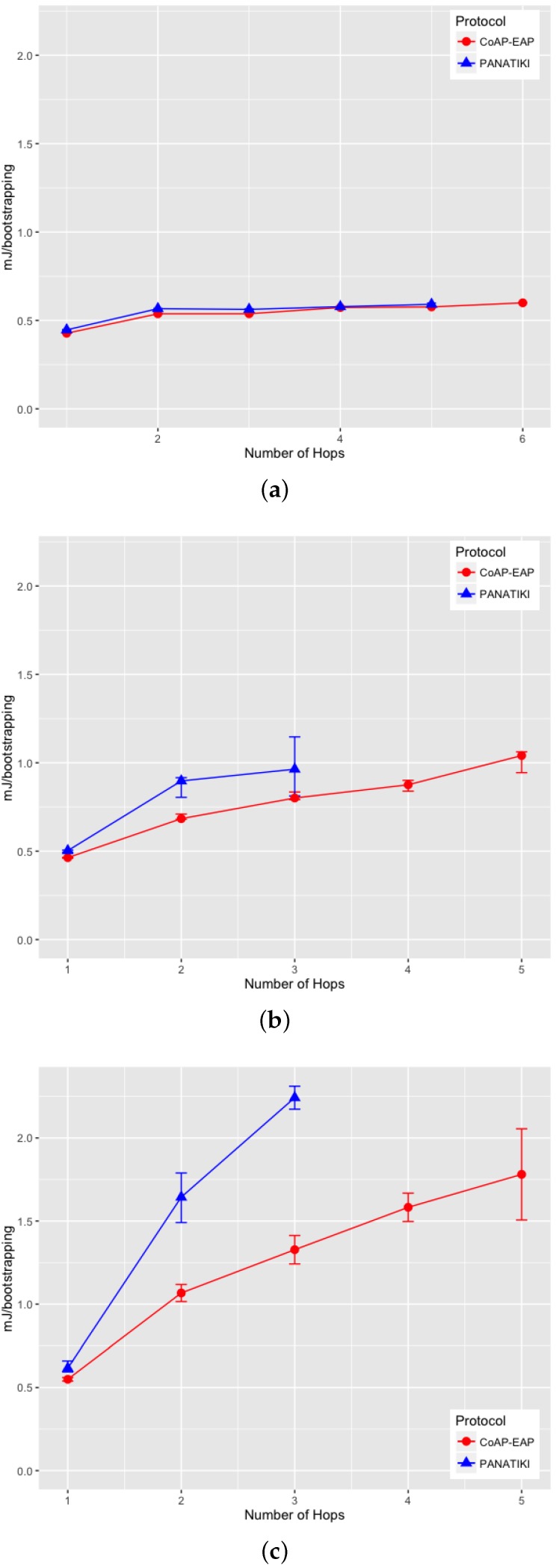
CPU energy consumption. (**a**) CPU energy consumption with 0.0 loss ratio; (**b**) CPU energy consumption with 0.1 loss ratio; (**c**) CPU energy consumption with 0.2 loss ratio.

**Figure 13:**
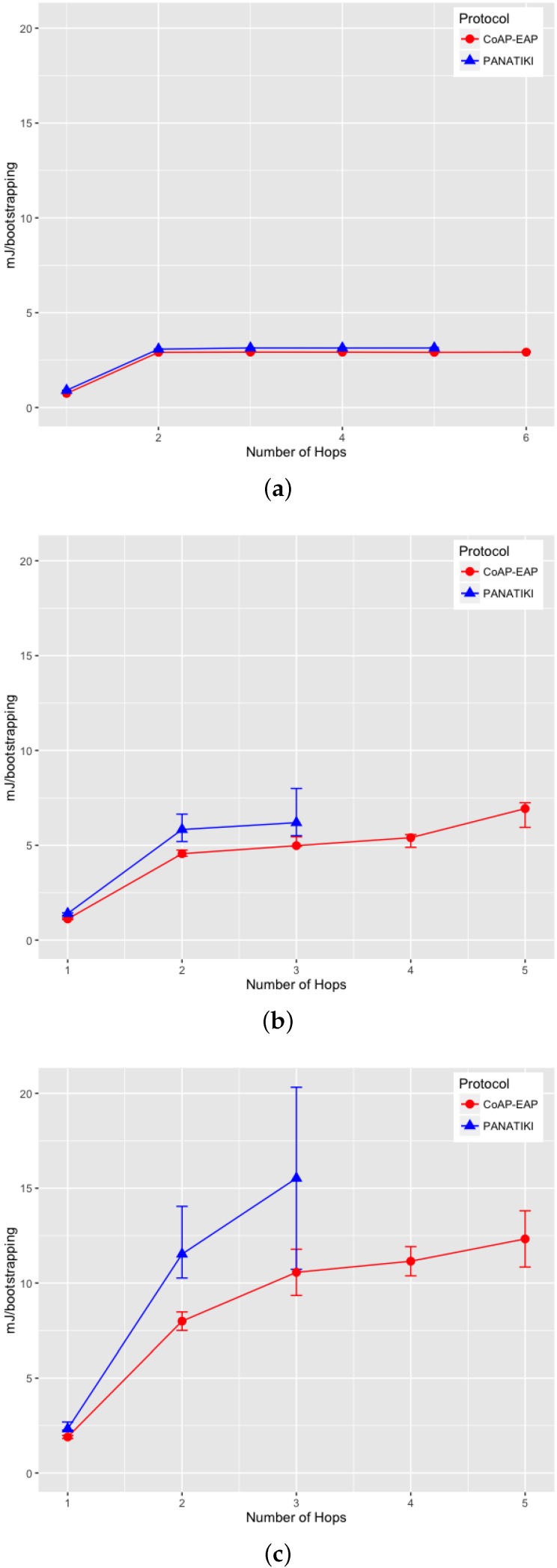
TX energy consumption. (**a**) TX energy consumption with 0.0 loss ratio; (**b**) TX energy consumption with 0.1 loss ratio; (**c**) TX energy consumption with 0.2 loss ratio.

**Figure 14:**
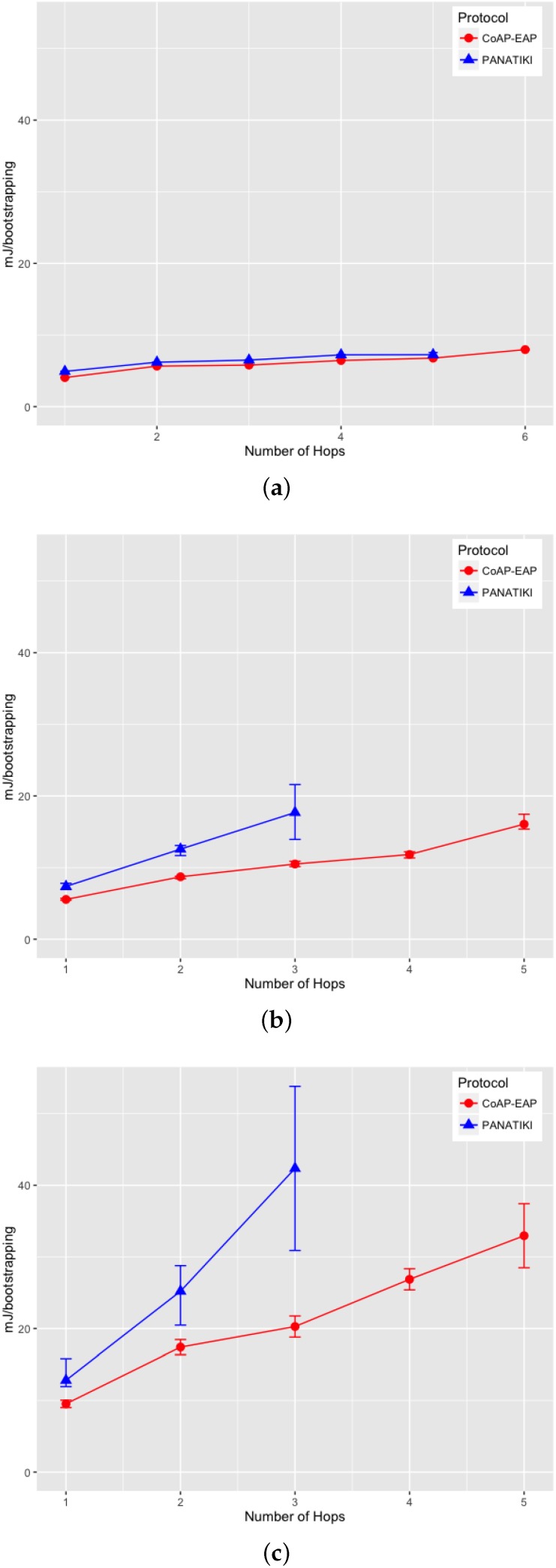
RX energy consumption. (**a**) RX energy consumption with 0.0 loss ratio; (**b**) RX energy consumption with 0.1 loss ratio; (**c**) RX energy consumption with 0.2 loss ratio.

**Figure 15:**
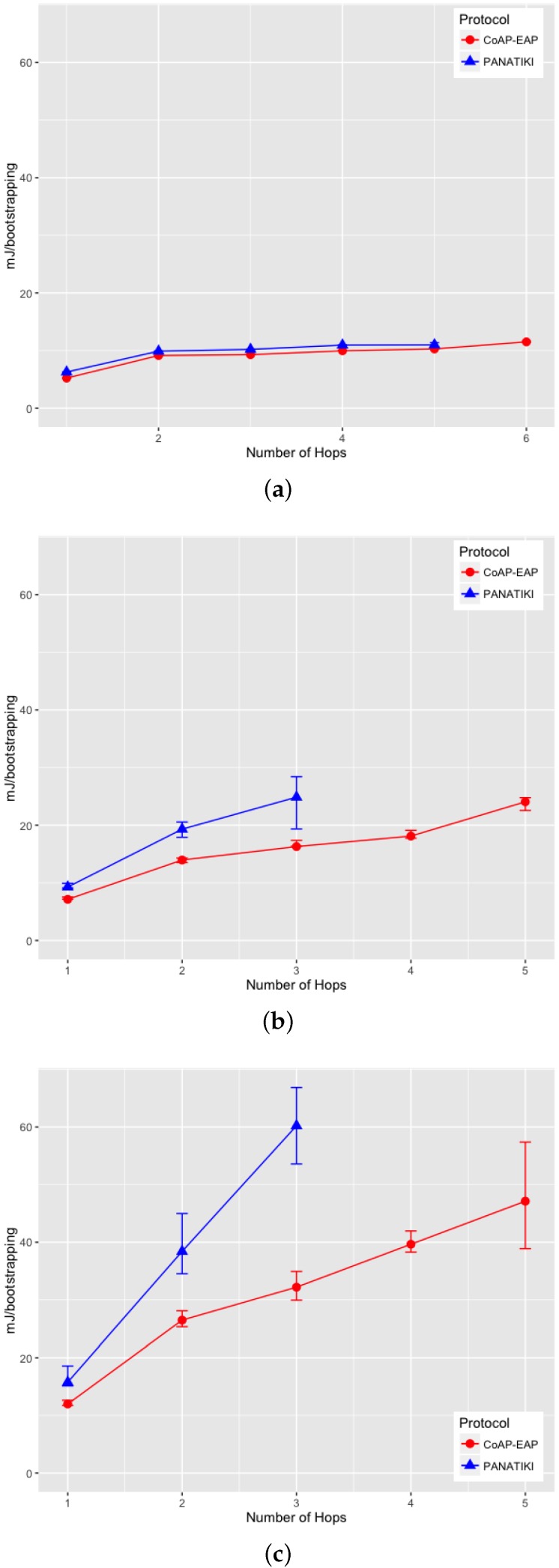
Total energy consumption. (**a**) Total energy consumption with 0.0 loss ratio; (**b**) Total energy consumption with 0.1 loss ratio; (**c**) Total energy consumption with 0.2 loss ratio.

To obtain the energy consumption, we used the Powertrace [105] tool that comes with Cooja simulator. We used it to estimate the median energy consumed by each bootstrapping (mJ/bootstrapping) in CoAP-EAP and PANATIKI. The different measurements show the CPU consumption when the Smart Object is fully operative; the consumption when transmitting (TX) and receiving (RX) consumption and, finally the total energy consumption of each solution. Figure 12a–c, Figure 13a–c, Figure 14a–c and Figure 15a–c show the energy consumption expressed in millijoules per bootstrapping (mJ/bootstrapping) in the same cases as Section 4.2.2.

As observed in all the measurements, as the packet loss ratio increases, more retransmissions (and, therefore, more message processing) are required (processing retransmitted messages, sending the retransmitted messages, *etc.*). Thus the energy spent because the CPU is working also increases. Additionally, we can see that the CPU consumption remains very similar in both alternatives when packet loss ratio is around 0. The reason is that the number of retransmissions is low and basically this CPU energy is spent on processing the messages shown in Figure 11. Since these values are very similar it is reasonable to observe similar CPU energy consumption. However, when the network conditions worsen, the CPU needs to work more in the case of PANATIKI than CoAP-EAP, which is an evidence that more retransmissions are required in PANATIKI due to more fragmentation as a consequence of the message length, and due to the more aggressive retransmission policy.

The energy spent transmitting (TX) (Figure 13a–c), as well as as receiving (RX) messages (Figure 14a–c) over the radio interface, are the most energy consuming tasks. In fact, both are more important than the energy consumed by the CPU.

If the circumstances are not adverse, the best case scenario with 1 hop and a packet loss ratio of 0, the CoAP-EAP behavior (≈5.2 mJ/bootstrapping) is slightly better than PANATIKI (≈6.3 mJ/bootstrapping). However, CoAP-EAP clearly reduces the energy consumption in contrast with PANATIKI when the network conditions are worse. For example, with 3 hops and 0.2 packet loss ratio PANATIKI spends around ≈60 mJ/bootstrapping in RX while CoAP-EAP spends ≈32 mJ/bootstrapping. This energy consumption is also evidence that PANATIKI involves more retransmissions and needs to send and, therefore, receive more messages.

In the majority of scenarios with a loss ratio greater than 0, CoAP-EAP proves to be significantly better in terms of energy consumption in each of the modes.

Finally, Figure 15a–c show the total energy consumption per bootstrapping including all these factors, where we can observe that CoAP-EAP proves to be a more energy-saving solution than PANATIKI.

## 5. Conclusions and Future Work

In this work we have discussed the importance of bootstrapping in IoT networks. We have presented a novel bootstrapping service that uses EAP, interacts with AAA infrastructures, and defines a new and simple EAP lower-layer using CoAP to provide a flexible, scalable, secure and constrained solution. After the bootstrapping is completed, we propose two ways of establishing a security association between the Smart Object and the Controller in an IoT network. The first uses a new CoAP Option (AUTH Option) defined for integrity protection and the second is done through DTLS. For this purpose a key hierarchy has been presented.

We have shown some performance results related to the message length, bootstrapping time and message processing time, memory footprint and energy consumption. We have contrasted these results with those obtained from PANATIKI, which is an optimized implementation for bootstrapping in IoT based on PANA. Therefore, it is good representative of other bootstrapping alternatives that use EAP and AAA and key technologies. We conclude that our solution, thanks to having a shorter message size brought to the overall bootstrapping process, such as bootstrapping time, probability of finishing the bootstrapping (success percentage) and energy consumption, substantial improvements. Other improvements such as memory footprint and message processing time are more limited, mainly due to the EAP method, even when EAP-PSK is considered lightweight. This suggests that more constrained methods may be required in the future. Nevertheless, when we focus on one of the key contribution of our work, the proposed CoAP-based EAP lower-layer, we observe that a part is substantially reduced in compared to PANA. In other words, we can conclude that the next steps in this field should be aimed at of improving or designing new EAP methods.

In this sense, future work has been planned for the use of other EAP methods (e.g., EAP-AKA [106]). Moreover, testing our bootstrapping service in *Low Power Wide Area Network*, where reducing the message size is vital, is under discussion. Finally, the next step is to study alternatives to provide authorization, key distribution and secure communications between smart objects from the same (or different) security domains *after* the bootstrapping (*post-bootstrapping*). This is related to how a smart object that successfully performed the bootstrapping and entered the security domain can perform different actions, in a secure and controlled fashion, and consume the services that are being offered by other entities.

## Figures and Tables

**Table 1:** Specifications of the computer used for the testbed.

Testbed PC	
CPU	Intel(R) Core(TM) i5-2400 CPU @ 3.10GH
RAM	4GiB DIMM DDR3 Synchronous 1333 MHz
O.S.	Ubuntu Server 12.04.5 LTS - 32 bits
Kernel	3.13.0-32-generic

**Table 2:** Message length.

CoAP-EAP			PANATIKI			% CoAP-EAP Reduction	
**Msg.**	**LL**	**LL+EAP**	**Msg.**	**LL**	**LL+EAP**	**LL**	LL**+EAP**
POST	13	13	PCI	16	16		
POST(nonce-c)	18	18	PAR	40	40		
ACK(nonce-s)	20	20	PAN	40	40		
POST(Req/Id)	17	22	PAR(Req/Id)	27	32		
ACK(Res/Id)	9	20	PAN(Res/Id)	25	36		
POST(EAP-PSK 1)	17	46	PAR(EAP-PSK 1)	27	56		
ACK(EAP-PSK 2)	9	69	PAR(EAP-PSK 2)	24	84 *		
POST(EAP-PSK 3)	17	76 *	PAR(EAP-PSK 3)	25	84 *		
ACK(EAP-PSK 4)	9	52	PAR(EAP-PSK 4)	25	68		
POST(EAP Success)	36	40	PAR(EAP Success)	84	88 *		
ACK	27	27	PAN	52	52		
**Total**	192	403		385	596	50.1%	32.4%

LL: lower-layer message length; LL+EAP: lower-layer message length including EAP message length; * This indicates the messages that produce fragmentation.

**Table 3:** Implementations memory size.

	Empty Main	Network Support (e.g., IP/UDP)	EAP	Lower Layer	Total Size
PANATIKI	62.7 kB	24.9 kB	9.4 kB	5.9 kB	102.9 kB *
CoAP-EAP	62.7 kB	24.9 kB	9.4 kB	3.8 kB (+4.6 kB cantcoap)	105.4 kB

* This size does not include any CoAP implementation.
